# Low profile REBOA device for increasing systolic blood pressure in hemodynamic instability: single-center 4-year experience of use of ER-REBOA

**DOI:** 10.1007/s00068-020-01586-9

**Published:** 2021-01-30

**Authors:** David T. McGreevy, Mitra Sadeghi, Kristofer F. Nilsson, Tal M. Hörer

**Affiliations:** 1 Department of Cardiothoracic and Vascular Surgery, Faculty of Medicine and Health, Örebro University, Örebro University Hospital, 701 85 Örebro, Sweden; 2 Department of Surgery, Faculty of Medicine and Health, Örebro University, Örebro, Sweden

**Keywords:** Vascular access, REBOA, Institution, Trauma, Shock, Endovascular resuscitation

## Abstract

**Background:**

Hemodynamic instability due to torso hemorrhage can be managed with the assistance of resuscitative endovascular balloon occlusion of the aorta (REBOA). This is a report of a single-center experience using the ER-REBOA™ catheter for traumatic and non-traumatic cases as an adjunct to hemorrhage control and as part of the EndoVascular resuscitation and Trauma Management (EVTM) concept. The objective of this report is to describe the clinical usage, technical success, results, complications and outcomes of the ER-REBOA™ catheter at Örebro University hospital, a middle-sized university hospital in Europe.

**Methods:**

Data concerning patients receiving the ER-REBOA™ catheter for any type of hemorrhagic shock and hemodynamic instability at Örebro University hospital in Sweden were collected prospectively from October 2015 to May 2020.

**Results:**

A total of 24 patients received the ER-REBOA™ catheter (with the intention to use) for traumatic and non-traumatic hemodynamic control; it was used in 22 patients. REBOA was performed or supervised by vascular surgeons using 7–8 Fr sheaths with an anatomic landmark or ultrasound guidance. Systolic blood pressure (SBP) increased significantly from 50 mmHg (0–63) to 95 mmHg (70–121) post REBOA. In this cohort, distal embolization and balloon rupture due to atherosclerosis were reported in one patient and two patients developed renal failure. There were no cases of balloon migration. Overall 30-day survival was 59%, with 45% for trauma patients and 73% for non-traumatic patients. Responders to REBOA had a significantly lower rate of mortality at both 24 h and 30 days.

**Conclusions:**

Our clinical data and experience show that the ER-REBOA™ catheter can be used for control of hemodynamic instability and to significantly increase SBP in both traumatic and non-traumatic cases, with relatively few complications. Responders to REBOA have a significantly lower rate of mortality.

## Introduction

Resuscitative endovascular balloon occlusion of the aorta (REBOA) is a rapidly evolving technique used to temporarily stabilize hemodynamically unstable patients by aortic occlusion. It is used in both traumatic and non-traumatic causes of truncal exsanguination [1–4]. The etiologies of shock that may require bedside or operative use of REBOA can be exsanguination from blunt or penetrating trauma; gastrointestinal- (GI), obstetric- and gynecology-derived bleeding events; visceral aneurysm rupture; thoracic and abdominal aortic aneurysm rupture; post-abdominal surgery; iatrogenic injuries; and, potentially, in cardiac arrest and other hemodynamic instabilities [5–8]. Örebro University Hospital in Sweden, a middle-sized university hospital in Europe, has been using aortic balloon occlusion and REBOA since the end of the 1990s and more recently as part of the EndoVascular resuscitation and Trauma Management (EVTM) concept [1, 9–13]. However, previous access-related complications due to the use of larger sheath sizes (≥11 Fr) have posed an obstacle for the use of REBOA [14, 15]. In October 2015, the Prytime ER-REBOA™ catheter (Boerne, TX, USA) was approved for use in the European Union (CE marked) and since then has been used, as well as other REBOA catheters, at Örebro University hospital.

The objective of this report was to describe the clinical use, technical success, results, complications and outcomes of the ER-REBOA™ catheter at a single center without the known limitations of international registry studies. The use of REBOA and the ER-REBOA™ catheter has not yet spread to the majority of European centers and this is the first report of its kind. This study is also unique in reporting all causes for ER-REBOA™ use at one center in a consecutive and prospective manner.

## Methods

The study was approved as part of the REBOA study data collection approval by the Regional Ethics Committee of Uppsala (study number: 2014/210; EPM, Uppsala, Sweden). This study is a retrospective analysis of prospectively collected data from Örebro University hospital, Sweden on the specific use of the ER-REBOA™ catheter for all causes by the EVTM group led by the vascular team. Örebro University Hospital is a regional referral hospital in Sweden and covers a county population of around 600,000 patients.

Inclusion criteria were the use of the ER-REBOA™ catheter from October 2015 to May 2020. Data were obtained retrospectively from patients’ journals concerning the cause of shock, vascular access technique, number of attempts, catheter migration or failure, zone and duration of occlusion, complications and clinical outcome. Medical records and data for all patients were analyzed by two doctors under the supervision of a senior vascular consultant. Balloon migration was defined as the unintentional distal migration of the REBOA more than 4 cm as seen on the catheter external markers or on fluoroscopy when available. Systolic blood pressure (SBP) measurements both pre- and post-REBOA inflation were retrieved from the medical and anesthetic records and the local REBOA registry database. Pre-REBOA SBP was the last measured value before aortic occlusion, post-REBOA SBP was the first measured value after aortic occlusion within 5 min. Based on post-REBOA SBP, patients were categorized as responders or non-responders, with a non-responder defined as a hypotensive patient with post-REBOA SBP <90 mmHg [16]. Regarding some variables, division of trauma and non-trauma patients was made as these patients significantly differ physiologically and no statistical comparison was made between the two.

### Statistical analysis

Continuous data are reported as median (range). Categorical and ordinal data are reported as numbers (percentages). Where data is missing, the valid percentages calculated from the available data are reported. Wilcoxon Signed-Rank test was used to compare the overall difference between two dependent groups with continuous data. Fisher’s Exact test was used to compare categorical data of the independent groups. Statistical significance was set at p-value < 0.05 (2-sided). Statistical analysis was performed using SPSS version 25 (IBM, Armonk, NY, USA).

## Results

During the 59-month study period, a total of 43 cases of REBOA using various REBOA catheters were reported at Örebro University Hospital, Sweden. Aortic balloon occlusion because of ruptured aortic aneurysms using other balloons were not included in this study. Two cases with intention to treat were excluded because the ER-REBOA™ was not inflated (one case became hemodynamically stable during resuscitation and, in one patient, there was a failure to advance in the iliac artery due to aortic-iliac injury). In both cases, the balloon was not inflated and was later withdrawn. Twenty-two cases were treated using the ER-REBOA™ catheter, 11 (50%) of them for traumatic causes of hemodynamic shock. Of the 11 traumatic cases, 5 (46%) were caused by blunt trauma and 6 (54%) by penetrating injuries (Table 1). The non-traumatic causes of shock were a result of GI bleeding (3, 27%), iatrogenic hemorrhage after cardiovascular procedures (3, 27%) or oncologic resection (1, 9%), obstetric hemorrhage (2, 18%), ruptured abdominal aortic aneurysm (1, 9%) and visceral aneurysm hemorrhage (1, 9%). The median age was 32 (IQR 26–73) years, with a majority of the patients being male (16, 73%). Most of the traumatic cases (10, 91%) were previously healthy whereas a majority of the non-traumatic cases (10, 91%) had several major comorbidities such as malignancy (5/11, 46%), cardiovascular (5/11, 46%), diabetes (4/11, 36%), anemia (2/11, 18%), COPD (1/11, 9%) and immunosuppression (1/11, 9%).

**Table 1:** Presented sources of bleeding in both trauma and non-trauma patients who received the ER-REBOA™ catheter for hemorrhagic shock at Örebro University hospital

Non-trauma
Colon ischemia
Iatrogenic injury to common iliac artery during urologic surgery
Ruptured abdominal aortic aneurysm
Emergent section
Placenta previa
Postoperative bleeding: duodenal ulcus
Postoperative bleeding: iliac artery after TAVI
Postoperative bleeding: PTA of superficial femoral artery
Postoperative bleeding: iliac artery after TEVAR
Rectal bleeding
Pseudoaneurysm hepatic artery
Trauma
*Blunt*
Pelvic fracture
Gastrointestinal bleeding: ileocolic artery
Massive long bone fractures + traumatic cardiac arrest
Hemopneumothorax + traumatic cardiac arrest
Ruptured spleen + traumatic cardiac arrest
*Penetrating*
Gunshot: common femoral artery and right lung
Axe to the head
Knife to abdomen: hemorrhage from pancreas
Knife to abdomen, thorax, neck
Gunshot: lung
Gunshot: gluteal

TAVI, transcatheter aortic valve implantation; PTA, percutaneous transluminal angioplasty; TEVAR, thoracic endovascular aortic repair

**Fig. 1:**
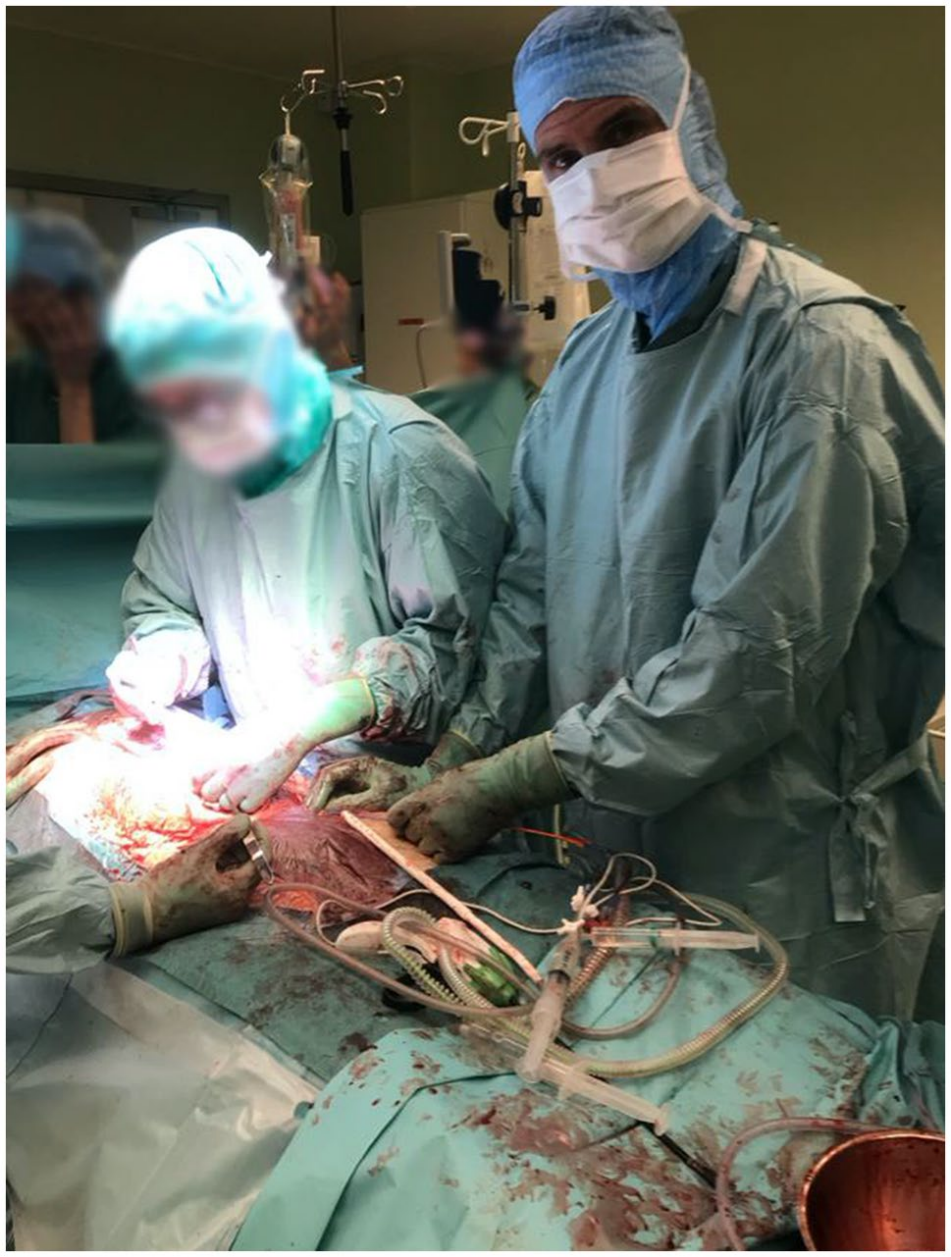
Placement of the ER-REBOA™ catheter in a hemodynamically unstable patient by a vascular resident. All residents participate in the EVTM and REBOA workshops. All procedures are supervised by a senior vascular surgeon. Note that the resident holds the ER-REBOA™ catheter at all times, adjusts and uses pREBOA if needed to support hemodynamic control. The peel-away sheath is also only retracted along the catheter and not removed, allowing the removal and re-use of the balloon later, if necessary, during the procedure

Vascular access, 7 Fr (12/22, 55%) and 8 Fr (10/22, 45%), was gained using landmark guidance in 6/18 (33%), ultrasound guidance in 11/18 (61%) and cut-down in 1/18 (6%). REBOA was performed by vascular surgeons, although in two cases the vascular access was performed by anesthesiologists under the guidance of a senior vascular surgeon. In four cases REBOA was performed by vascular residents supervised by a senior vascular surgeon (Fig. 1). In the majority of the traumatic cases, vascular access was established in the emergency room (ER) (9/10, 90%). In the non-traumatic cases, vascular access was established in the operating room (OR) or angiography suite (AS) in 7/11 (64%) of cases, in the ER in 3/11 (27%) of cases and in the intensive care unit (ICU) in 1 (9%) case. Of the 12 cases where vascular access was performed in the ER, 9/12 (75%) were taken to the OR, 1/12 (8%) were taken to the angio suit and 2/12 (17%) died in the ER after CPR. In all cases, vascular access was placed in the common femoral artery and closed by manual compression or closure device (Angioseal, Terumo).

**Fig. 2:**
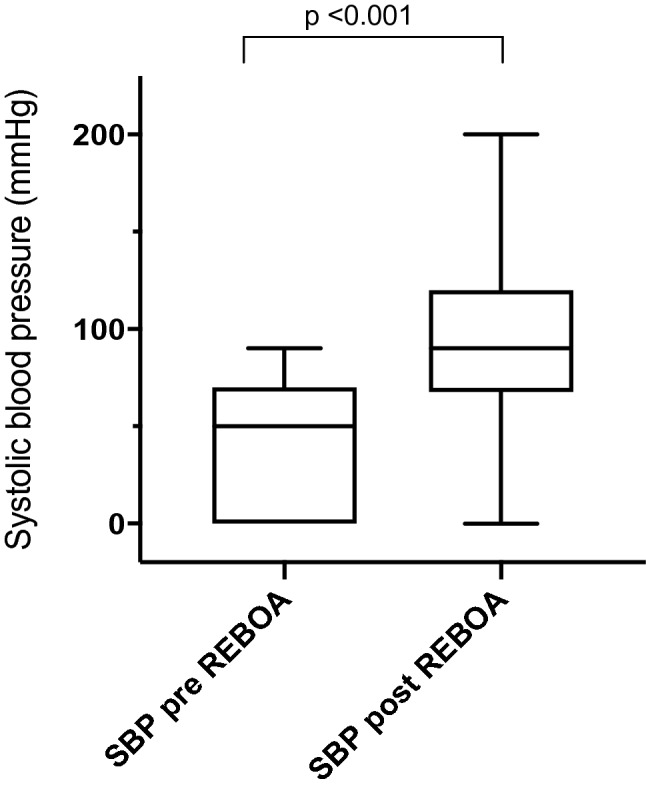
Systolic blood pressure in patients before and after receiving an ER-REBOA™ catheter for hemodynamic instability

The median pre-REBOA SBP was 50 mmHg (0–63) (Table 2). Median overall post-REBOA SBP significantly increased to 95 mmHg (70–121) (*p* < 0.001) (Fig. 2). An improvement in SBP was seen between pre-REBOA SBP and post-REBOA SBP in 16 patients, with a mean increase of 60 mmHg (15–120). In the remaining 6 patients there was missing data regarding post-REBOA SBP of whom 3 died in the ER during CPR and 3 survived post 30-days, none of whom received CPR. In one patient with asystole and complete hemodynamic collapse there was no change in the SBP during REBOA. Ten (53%) patients were classified as REBOA responders. The balloon was used in zone I in 9/10 (90%) and zone III in 1/10 (10%) of traumatic cases, and in zone I in 6/11 (55%) and zone III in 7/11 (64%) of non-traumatic cases. The median total inflation time was 38 min (22–59). Total occlusion was reported at some stage of treatment in all cases (100%), but both total and partial occlusion were documented in 6/20 (30%) of cases. Adjustment of balloon position within zones was made in 4/20 (20%) of cases.

**Table 2:** SBP, CPR and ROSC pre- and post-REBOA, occlusion time and type, complications and outcome of 22 patients with traumatic and non-traumatic hemodynamic shock receiving the ER-REBOA™ catheter

Variables	Total
SBP pre REBOA, median mmHg (IQR)	50 (0–63)
SBP post REBOA, median mmHg (IQR)	95 (70–121)
CPR, *n* (%)	
Pre REBOA	8 (40)
During REBOA	4 (22)
ROSC, *n* (%)	
Pre REBOA	6 (75)
Post REBOA	5 (29)
Total occlusion time, median min (IQR)	38 (22–59)
Occlusion type, *n* (%)	
Total occlusion	22 (100)
Partial occlusion	6 (30)
Outcome	
24-h survival, *n* (%)	15 (68)
Hospital stay, median days (IQR)	6 (1–28)
30-day survival, *n* (%)	13 (59)

SBP, systolic blood pressure; IQR, interquartile range; CPR, cardiopulmonary resuscitation; ROSC, return of spontaneous circulation; REBOA, resuscitative endovascular balloon occlusion of the aorta

During resuscitation with REBOA, 18/22 (82%) of patients received transfusion using a mean of 6.8 units of red blood cells, 4.8 units of fresh frozen plasma and 1 unit of platelets.

### Cardio-pulmonary resuscitation (CPR)

Prior to REBOA insertion, CPR had been performed in 8 (40%) cases; return of spontaneous circulation (ROSC) pre-REBOA was observed in 6 (75%) of these. REBOA was performed during CPR in 4 (22%) cases and ROSC post-REBOA was observed in 3 (75%) of these. Of the patients who had CPR and ROSC pre-REBOA, 2/6 (33%) survived post 24 h. Of the 4 cases where REBOA was performed during CPR, 2/4 (50%) survived post 24 h and 1/4 (25%) survived post 30 days.

### Complications

Overall complications, both procedural and organ related, were reported in 14% of patients and in 20% of those who survived post 24 h. One (5%) of the trauma patients suffered distal embolization caused by the vascular access, which was resolved by surgical embolectomy, and two (10%) trauma patients were reported to have developed renal failure with renal function improving over time. None of the non-trauma patients suffered from distal embolization or renal failure. There were no reported cases of access to bleeding or multiple organ failure (MOF). Balloon rupture occurred in one (5%) non-trauma, non-CPR, GI elderly patient with extensive atherosclerosis; a replacement balloon was then used. Balloon migration was not recorded in any of the cases.

### Clinical course and outcome

Patients stayed in the hospital for a median of 6 days (1–28). Of the traumatic cases, 5/11 patients (45%) survived post 24 h. Of the 6 patients who died, 4 (67%) deaths were due to hemorrhage. Ten (91%) patients survived post 24 h in the non-traumatic cases. The 30-day survival rate was 59% for all patients; 45% for traumatic cases (with no difference between blunt or penetrating causes of hemorrhage) and 73% for non-traumatic cases. Those patients who were responders to REBOA had a significantly lower rate of mortality at both 24 h and 30 days (Table 3).

**Table 3:** Univariate analysis comparing clinical data of 22 patients of which 10 were REBOA responders and 9 were REBOA non-responders

Mortality	All (*n* = 22)	Responders (*n* = 10)	Non-responders (*n* = 9)	p*
Mortality 24 h	7 (32%)	0 (0%)	7 (78%)	< 0.001
Mortality 30 days	9 (41%)	1 (10%)	8 (89%)	0.001

Data are presented as number (%). Numbers may not add up to 22 because there were missing values. The presented percentages are the valid percentages calculated from available data. *p = Fisher’s Exact test

## Discussion

This is a single-center report on the use of the ER-REBOA™ catheter for hemorrhage control and resuscitation in line with the EVTM concept. It is, to the best of our knowledge, the first known consecutive series of patients in Europe where an ER-REBOA™ catheter was used [17]. Using the ER-REBOA™ catheter in a total of 22 patients, our results show an overall survival rate of 59% in patients with severe hemorrhagic shock, a 25% survival rate in those patients where CPR was performed during REBOA and a 90% survival rate in REBOA responders. The survival rate was numerically higher among non-traumatic patients (73%) compared to trauma patients (45%), despite the higher rate of comorbidities for non-traumatic patients. One hypothetical reason for this could be that non-traumatic patients were in the hospital and under medical observation with fast involvement of the vascular EVTM team and time-to-REBOA was, therefore, shorter compared to trauma patients.

The ER-REBOA™ catheter caused a significant increase in SBP in the majority of patients and balloon rupture only occurred in one case due to severe atherosclerosis, while no cases of balloon migration were found in this cohort. Vascular access complications were rare. Recent publications from our group and others show a low rate of access complications and therefore confirm these results [8, 9, 11]. We have learnt from our clinical experience to always fixate the vascular access using a simple suture, to note the position of the balloon using the external length markers and, at all times, to have a dedicated person in control of vascular access, balloon position and inflation/deflation in response to the hemodynamic status of the patient.

The ER-REBOA™ catheter has gained popularity due to several factors, such as being a low profile device (7 Fr) and having an atraumatic tip for optimized use in the emergency setting. The procedure has been simplified with external length markers to assist the positioning of the device and without the need for a guidewire or fluoroscopic placement verification, allowing quicker hemodynamic control and decreasing the risk for complications. A recent paper by Brenner et al. [18] describes the outcome of trauma patients receiving the ER-REBOA™ catheter, concluding that these low profile devices are effective at giving a significant increase in SBP and also a higher rate of ROSC (68%) compared to their earlier experience. In our study, most patients receiving CPR had ROSC before REBOA was used. Four patients, however, later re-arrested and received REBOA during CPR, 3 (75%) of whom achieved ROSC after balloon inflation, with 2 patients (50%) surviving post 24-h and 1 patient (25%) surviving post 30 days. Moore et al. previously reported a 0% survival rate for patients in arrest at the time of REBOA [19]. Recent clinical and experimental research are investigating the role of REBOA as an adjunct during CPR, suggesting that this cohort of patients could potentially benefit from REBOA in resuscitation efforts [20]. With REBOA supporting cardiac and cerebral perfusion, one may speculate that its use may help prevent more patients from re-arresting and might suggest a benefit of early use, however, a randomized controlled trial is needed. Our results also support previously published data by Duchesne et al. that REBOA non-responders died more frequently than did REBOA responders [16]. The ER-REBOA™ catheter allows the possibility of SBP monitoring proximal to the balloon and combined with sheath SBP measurements facilitates monitoring of hemodynamic response to aortic occlusion and verification of partial (pREBOA) and intermittent occlusion (iREBOA), if used. A major benefit of pREBOA is the possibility to titrate the blood pressure during preparation for definitive treatment.

Brenner et al. also concluded that the low-profile device does not reduce the need for surgical cut-down to gain femoral access [15]. Based on our own experience, ultrasound-guided percutaneous access can be performed with a high primary success rate, despite a depleted circulation or ongoing CPR [8]. With adequate training, the use of an ultrasound-guided technique has been shown to reduce procedure time and minimize complications as seen in the low rate of complications in this material [21]. The additional benefit of ultrasound access and use of REBOA catheters compatible with smaller sheath sizes is that surgical repair is not mandatory since a closure device can be used [13].

Overall complications, both procedural and organ related, are reported in our study as 14%. This complication rate may partly be a result of some patients dying within 24 h, with the complication rate in those surviving post 24 h being 20%, but it is also likely related to the use of low profile devices allowing improved distal arterial flow, and an increased awareness of access site complications [14]. At our institution, we aim to directly place a 7–8 Fr sheath without upgrading from a smaller sheath if REBOA use is urgent, with no increase in reports of access complications. However, if the need for REBOA is uncertain we routinely place a femoral arterial line catheter (4–5 Fr) allowing the possibility to rapidly upsize if needed. Additionally, at our institution we strive to use pREBOA if hemodynamically possible, used in 30% of cases in this report, which decreases the risk of ischemic injury to internal organs and associated complications [11]. When possible, pREBOA is carried out by performing initial total aortic occlusion and then slowly deflating the balloon to achieve a below balloon mean arterial pressure (MAP) of 30 mmHg; however, due to the nature of this urgent procedure, data reporting is challenging and a firm conclusion cannot be drawn.

In the 6 cases of REBOA use in penetrating trauma, all but one received their vascular access and REBOA in the ER and where quickly transported to the OR. In the OR, 3 cases where found to have multiple sources of bleeding in both the abdominal and thoracic cavities, one case of isolated abdominal bleeding from the pancreatic artery and one case of penetrating head trauma. As vascular access and REBOA placement at Örebro University hospital are performed according to the EVTM concept, by physicians who are highly trained in this area, no delay to the initial primary survey and following transportation to the OR is estimated.

All procedures reported in this article were performed by physicians who had attended the EVTM workshops and were highly trained in vascular access and REBOA [13]. This might also explain the relatively low rate of vascular access complications and MOF due to the extensive use of short occlusion time, pREBOA and other EVTM methods. EVTM aims to combine modern endovascular techniques and procedures with traditional Advanced Trauma Life Support (ATLS) and Definitive Surgical Trauma Care (DSTC) for early multidisciplinary evaluation, resuscitation and definitive management of hemodynamically unstable patients. This concept does not replace traditional surgical or other solutions but instead incorporates all available tools into a common trunk. The application of EVTM is highly dependent on the skillset and capabilities of the managing team or center and the resources available, therefore can and should be modified accordingly. It has been suggested that using the EVTM concept may result in faster bleeding control, minimized blood loss, and less extensive surgical insult. It may also assist surgical teams assemble necessary resources and saving precious time [13, 20, 22, 23].

### Limitations

First, this report is limited by the small number of included patients and the lack of a control group, it is therefore a descriptive study and observations should be interpreted cautiously. This can, however, despite the small number of patients also be seen as a strength of the study as it is a single center cohort reporting “real world” data of using the ER-REBOA™ catheter without the known limitations of international registry studies and with little missing data. Secondly, trauma-related deaths often occur within 24 h of the injury which does not allow the development of MOF and might also reduce the number of notifications of complications related to vascular access.

## Conclusions

Our clinical data and experience show that the ER-REBOA™ catheter can be used to control hemodynamic instability and to significantly increase SBP in both traumatic and non-traumatic cases, with relatively few complications. Responders to REBOA have a significantly lower rate of mortality. However, it is important to remember that this is a limited cohort of patients and further larger studies are needed.

## Data Availability

Software application.

## Availability of data and material

Available upon request.
